# Supplementary material for: Validation of the Champion Health Belief Model Scale for an Investigation of Breast Cancer Screening Behaviour in Malaysia
Source: Int J Environ Res Public Health. 2021 Sep 3;18(17):9311. doi: 10.3390/ijerph18179311 (PMC8431600; doi:10.3390/ijerph18179311)
Supplement: Supplementary file 1 [file ijerph-18-09311-s001.zip › ijerph-1329312-supplementary.pdf]

Supplementary Table

Supplementary Table S1. Eigenvalues and the percent of variance explained by subscales in the factor analysis.

**Factor**	**Initial Eigenvalues**			**Percentage of variance explained by each subscale**	
	**Total**	**Percentage of variance**	**Cumulative percentage**	**Percentage of variance**	**Cumulative percentage**
1	5.28	25.13	25.13	22.08	22.08
2	2.32	11.04	36.17	9.21	31.28
3	2.16	10.26	46.43	7.57	38.86
4	1.21	5.76	52.19		
5	1.13	5.36	57.55		
6	0.99	4.69	62.24		
7	0.90	4.29	66.53		
8	0.86	4.11	70.64		
9	0.74	3.53	74.17		
10	0.66	3.16	77.34		
11	0.61	2.91	80.24		
12	0.57	2.74	82.98		
13	0.55	2.62	85.60		
14	0.51	2.44	88.04		
15	0.45	2.12	90.16		
16	0.44	2.10	92.26		
17	0.40	1.89	94.16		
18	0.37	1.74	95.89		
19	0.33	1.56	97.46		
20	0.29	1.36	98.82		
21	0.25	1.18	100.00		
